# Internet of Things (IoT)-Enhanced Applied Behavior Analysis (ABA) for Special Education Needs

**DOI:** 10.3390/s21196693

**Published:** 2021-10-08

**Authors:** Chun Man Victor Wong, Rosanna Yuen-Yan Chan, Yen Na Yum, Kangzhong Wang

**Affiliations:** 1 Department of Special Education and Counselling, The Education University of Hong Kong, Tai Po, N.T., Hong Kong, China; s1138617@s.eduhk.hk (C.M.V.W.); yyum@eduhk.hk (Y.N.Y.); 2 Centre for Perceptual and Interactive Intelligence, The Chinese University of Hong Kong, Shatin, N.T., Hong Kong, China; 3 Department of Information Engineering, The Chinese University of Hong Kong, Shatin, N.T., Hong Kong, China; wongkz@link.cuhk.edu.hk

**Keywords:** Internet of Things (IoT), applied behavior analysis (ABA), autism spectrum disorder (ASD), special education needs (SEN)

## Abstract

Applied behavior analysis (ABA) has become a popular behavioral therapy in the special education needs (SEN) community. ABA is used to manage SEN students’ behaviors by solving problems in socially important settings, and puts emphasis on having precise measurements on physical and observable events. In this work, we present how Internet of Things (IoT) technologies can be applied to enhance ABA therapy in normal SEN classroom settings. We measured (1) learning performance data, (2) learners’ physiological data, and (3) learning environment sensors’ data. Upon preliminary analysis, we have found that learners’ physiological data is highly diverse, while learner performance seems to be related to learners’ electrodermal activity. Our preliminary findings suggest the possibility of enhancing ABA for SEN with IoT technologies.

## 1. Introduction

Applied Behavior Analysis (ABA) aims at the scientific understanding and improvement of human behavior. ABA therapy is becoming a popular behavioral therapy for children with special education needs (SEN) such as autism spectrum disorders (ASD) [1]. Despite well-documented evidence that ABA therapy is effective in fostering the growth and development of students with SEN in their behavior, communication, social interaction, self-help, play, and academic skills [2], support and therapy for students with SEN have always been unequally distributed. One of the reasons is that ABA therapy requires highly intensive treatment, case supervision, and caregiver training [3], making it affordable by only those from high-income families. Therefore, accessible and affordable options for ABA therapy is an important goal in inclusive education.

The Internet of Things (IoT) and the related Wireless Body Area Network (WBAN) solutions are very promising for patient monitoring in healthcare systems, such as those presented in the current Special Issues. Relatively less work has been done on applying IoT to enhance educational technologies which target students with SEN. There exists an intersecting set of design goals between IoT and desirable systems which support ABA therapy, such as very low latency (on the order of 1 ms), human-to-human (H2H)/machine-to-machine (M2M) coexistence, and integration of Wi-Fi based data-centric technologies [4]. The design and implementation of IoT-enhanced educational systems for ABA therapy require an interdisciplinary understanding between technologies and SEN. This yields only a little success in integrated solutions in IoT for SEN so far.

Our cross-disciplinary research team in this study comprised of members from communications engineering and special education. The first author, who comes from the field of ABA therapy, designed the Integrated Intelligent Intervention-learning system (3i-learning system) based on his frontier experiences. The system aims at providing AI-empowered personalized learning for ABA therapy, so that conventional ABA therapy instructions can be delivered in the form of personalized learning objects via the mobile client of an intelligent system (the Bridge AI^TM^ system). Furthermore, the system is augmented by IoT technologies, namely those for sensing participants’ physiological conditions and the surrounding classroom environment. Through dynamically feeding the learning measurement data into the system, an extensive view of the therapy progress for each child can be produced via (1) measurements of task performances, and (2) environmental and (3) physiological factors that affect the child’s learning.

### Our Contributions and Significance

In this work, we designed and implemented an ABA therapy system that was enhanced by IoT technologies. We performed a user study with 15 students (all having autism spectrum disorders and moderate intellectual disabilities) and 10 teachers from a special school in Hong Kong. In addition to an actual improvement in students’ adaptive behaviors, individual differences in students’ physiological biomarkers, as well as statistically significant relationships between IoT sensor readings and learning-related indicators, were also identified. Our work is amongst the pioneering attempts (along with, for example, [5,6,7]) in improving ABA therapy with sensor technologies. It provides an important reference case of sensors and biomedical signal processing for patient monitoring in IoT literature.

## 2. Theoretical Background

### 2.1. Autism Spectrum Disorders (ASD)

Autism spectrum disorder (ASD) is a neuro-developmental disorder characterized by persistent deficits in social communication and interactions, together with restricted interests and repetitive patterns of behaviors [8]. The prevalence of childhood ASD in the Greater China Region is 26.6 per 10,000 children [9], with increasing figures in recent years due to the expansion of diagnostic criteria and a higher public awareness. The clinical presentations of ASD vary to a large extent, and approximately 56% of children with ASD possess below average intellectual ability (IQ < 85) [10]. A characteristic of ASD is abnormal response to sensory stimulation, which can manifest as extreme sensitivity to light or sound (hypersensitivity), or decreased response to stimuli (hyposensitivity). A recent study reported increased internal noise and worse external noise filtering in individuals with ASD [11]. This points to the importance of accounting for environmental influence when working with children with ASD. The intellectual and verbal abilities, as well as differences in sensory processing, in this population may directly influence intervention outcomes; however, the extant literature lacks systematic studies on the relationships of these variables.

Treatment directions may include management of self-control issues or challenging behaviors and addressing the core social deficits in ASD [12]. Reducing undesirable behaviors and developing social skills of at-risk school-aged children with ASD may mitigate bullying cases and increase the likelihood of social inclusion into the community. The involvement of parents or caregivers in interventions can give consistent expectations to students in home and school environments, which may increase intervention effectiveness. These improvements would likely increase mental health and well-being for individuals with ASD and their caregivers. The field has made strides in the early diagnosis of ASD, and there is still no evidence-based cure or pharmacological intervention for ASD.

The main method used to support individuals with ASD with integration into school and society remains behavioral interventions. Early research on ASD interventions has focused on comprehensive behavioral management based on the principles of behavioral modification, such as applied behavioral analysis (ABA). Such interventions are well-established to be effective when used individually and intensively for a prolonged period. However, resource limitations may prevent their wide and sustained use or, alternatively, lead to long wait times before intervention at publicly funded institutions.

### 2.2. Applied Behavioral Analysis (ABA)

Applied Behavioral Analysis (ABA) is an approach integrated as a core skill in applied and health psychology programs, and is considered a practice in psychology that is well-grounded in psychological science and evidence-based practice [13]. Granpeesheh et al. [14] presented the effectiveness of ABA in children with ASD. ABA therapy has demonstrated significant results in multiple areas, including academics, social functioning, independent living skills, vocational skills, challenging behaviors, and feeding disorders. This clearly exhibits how extensive ABA therapy is in aiding and supporting the growth and development of children with SEN.

On top of this, the results achieved through consistent ABA therapy are not limited to children with ASD. The research field of behavioral treatments has also looked into the effectiveness of ABA therapy in helping children with Attention Deficit Hyperactivity Disorder (ADHD). Fabiano et al. (2009) [15] summarized over 100 independent research studies, and the results indicate the effectiveness of behavioral treatments in improving the functioning of students with ADHD. The success of behavioral interventions such as ABA therapy are also researched and demonstrated in groups of children with other intellectual or developmental disorders such as intellectual disability (Hassiotis et al., 2011 [16]) and Down syndrome (Feeley & Jones, 2008 [17]), making ABA therapy a viable option for children with different SEN.

### 2.3. Affective Computing for Education

Affective computing is a hot topic with an ever-increasing popularity, which is mainly driven by its promising application in numerous areas [18]. According to Picard [19], the original definition of affective computing refers to computing that relates to, arises from, or deliberately influences emotion or other affective phenomena. In his book, the tasks of affective computing can be identified as two types: (1) detecting and recognizing subjects’ emotional information, and (2) empowering machines with emotional intelligence (e.g., perceiving and expressing their emotion states, like humans). The advent of affective computing literally marks the beginning of the exploration of recognition systems for human affects, and creates more possibilities to achieve effective human–computer interaction.

Recent studies have shown that affection can significantly influence the learning state of learners [20,21]. For example, affective lessons can arouse students’ targeted emotions and increase learning engagement [22,23]. Cognitive processing and affective function influence a learner’s attitude and motivation when using multimedia learning materials [24]. In the research conducted by Plass et al. [25], it was observed that positive emotions induced by the learning environment can result in better comprehension performance and transfer performance in students. Hence, the teaching quality and teaching strategies can be improved if teachers are capable of perceiving and inducing students’ positive affective states, which can, in turn, enable effective and efficient learning.

In view of the development of physiological sensor technologies, affective computing techniques can be successfully applied in education systems and help observe, monitor, and make responses to the changes in students’ emotions. Much research in affective computing in education mainly focuses on detecting affective states using physiological data (e.g., Blood Volume Pulse [BVP] and Electrodermal Activity [EDA]) collected from various sensors. In addition to physiological signals, other bio-features contained in human communications, such as facial expressions, gestures, speech, and verbalization can also be used in building effective affection recognition systems. For example, Lin et al. [26] proposed an affective tutoring system that can capture and recognize learners’ emotional expressions during the learning process. Their system allows interactive agents to choose the most appropriate teaching strategies and complete animation feedback to users, which enables learners’ motivation, usability, and interaction. Affective computing will become one of the most powerful and effective tools to facilitate more efficient and smarter education.

## 3. Integrated Intelligent Intervention-Learning System (3i-Learning System)

Based on the solid theoretical background reviewed above, the research team gathered experiences from professionals in the field of ABA therapy and developed the Integrated Intelligent Intervention-learning system (3i-learning system). The system aims to train up parents and caregivers to become therapists of their children. The integration between technology and humanity is two-fold:

- The first integration that the 3i-learning system brings, through providing abundant training, is that parents and caregivers are integrated into the education plan of children as therapists, and they will be equipped with the knowledge and techniques necessary to carry out therapy sessions with their children using the system;
- The second integration that the 3i-learning system brings lies in the field of technology. Through integrating Artificial Intelligence (AI) and Internet of Things (IoT) measurements into the 3i-learning system, users will be provided with an extensive view of the progress of therapy for each child via measurements of task performance, as well as environmental and physiological factors that affect the child’s learning.

The 3i-learning system is also capable of assisting users in creating therapy plans to maximize progress and development with the help of the system AI engine. There are three core functional components in our system, namely:

1. Student-centered learning environments (SCLEs);
2. Learning objects (LOs);
3. Emotion recognizer.

**Figure 1:**
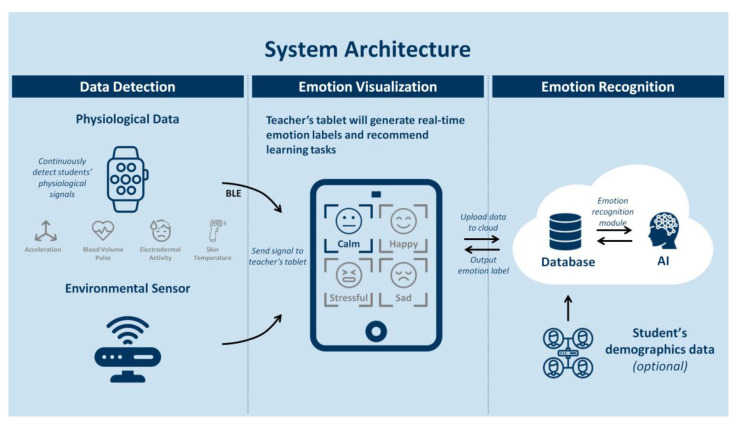
Overall system architecture of the 3i-learning system.

Each of these will be elaborated upon further in the current section. The overall system architecture of our system is provided in Figure 1.

### 3.1. Student-Centered Learning Environments (SCLEs)

The student-centered learning environment (SCLE) is a constructivist instructional design framework [27]. By enhancing learning tasks with technologies, resources, and scaffolding, students are expected to engage in personalized sense-making activities more productively [28]. There are four primary subcomponents in the SCLEs maintained by our system, namely:

- *Contexts*, which refer to the nature of the overall ABA learning tasks;
- *Tools*, which are various learning technologies, including the IoT devices, used to enrich the learning environments (i.e., the classroom);
- *Resources*, which correspond to the learning contents delivered via an Android mobile client;
- *Scaffolds*, that are the support mechanisms provided to the student to conduct his/her personalized learning tasks.

**Figure 2:**
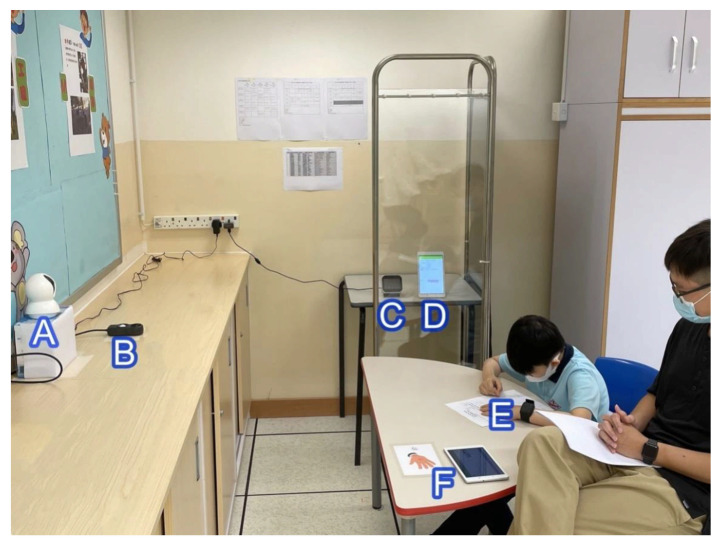
The SEN classroom serving as the context of our student-centered learning environment where IoT-enhanced ABA was delivered. (A) Camera; (B) Light Sensor; (C) CO_2_/Humidity/Temperature Sensor; (D) Emotion Recognizer; (E) E4 wristband; (F) mobile client of the 3i-Learning System.

**Figure 3:**
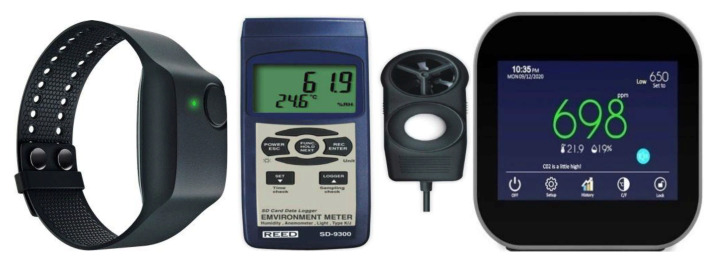
The E4 Wristband(**left**), REED SD-9300 Data Logging Environmental Meter (**middle**), and GZAIR Model 2 Indoor CO_2_ Meter (**right**).

The SEN classroom (as the context of our SCLE) and IoT devices (as tools delivering the relearning sources and scaffolds) are provided in Figure 2 and Figure 3, respectively.

### 3.2. Learning Objects (LOs)

**Figure 4:**
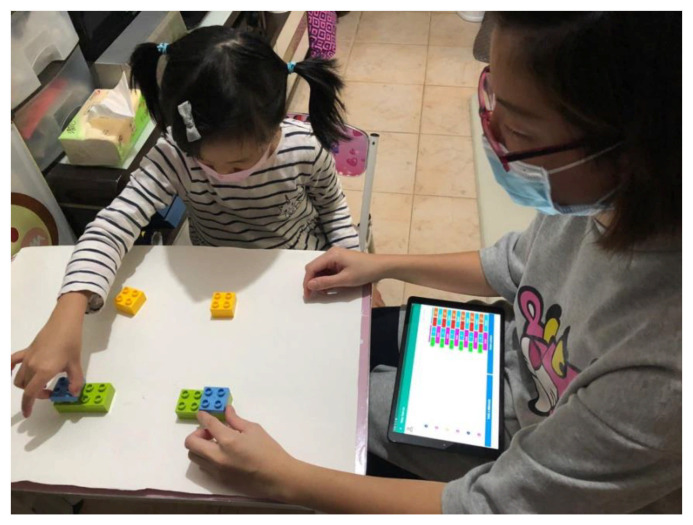
Modularized learning object (LO) in physical format (left) and in digital format delivered by the 3i-learning system mobile client interface (right).

According to the constructivists’ perspective [29], learning can be more motivational and meaningful to the student when the resources (i.e., the learning content) can be represented by physical or digital objects [30] called learning objects (LOs). LOs can be used to support highly personalized learning, which is a good instructional design option for IoT-enhanced ABA lessons. The main feature of LOs is that they are highly modularized and can divide the whole lesson into small chunks so that they can be reused multiple times in different learning contexts [31]. A typical LO delivered via the application client interface of our 3i-learning system is shown in Figure 4 below.

### 3.3. Emotion Recognizer

The emotion recognizer consists of various IoT-based data collection devices and an AI-engine capable of emotion recognition. During an ABA session, an Empatica E4 wristband [32] continuously detects and transmits students’ physiological signals including:

- Blood Volume Pulse (BVP);
- Acceleration (ACC);
- Electrodermal Activity (EDA) which is measured as galvanic skin response (GSR);
- Skin Temperature (SKT).

These signals are sent to the Android mobile client (which serves as our emotion recognition application) on the teacher’s tablet in the form of Bluetooth Low Energy (BLE) signals. These data are simultaneously uploaded to the cloud server.

**Figure 5:**
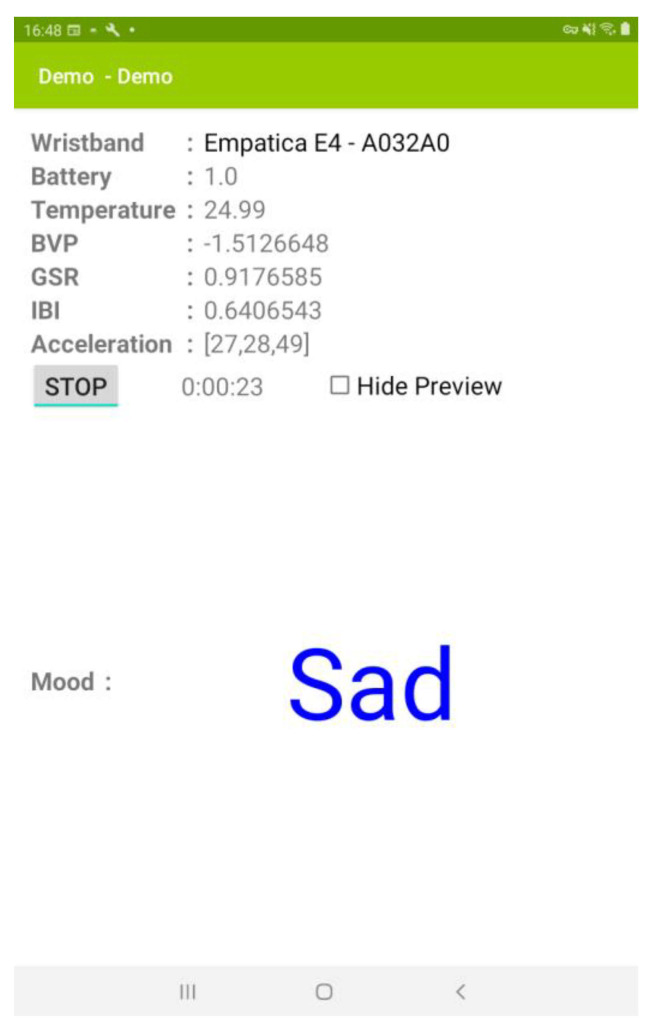
Emotion visualizer of the 3i-learning system.

Once the student’s physiological data is uploaded, our emotion recognition module, which resides in the cloud, executes the emotion inference task and outputs the corresponding emotion’s label. Furthermore, the real-time emotion labels generated by the emotion recognition module (together with the recommended learning tasks) are sent to the teacher’s tablet. The emotion recognizer is illustrated in Figure 5.

## 4. Data Collection

In this section, we present the data that was collected in the experiment setting. Detailed learning analytics are included in the next section, where we discuss the system evaluation in detail.

### 4.1. Learning Environment Data

We collected the light intensity, level of CO_2_, humidity, and temperature inside the classroom. We measured these values because they are known environmental factors affecting students’ learning [33], especially students with disabilities [34]. The data are summarized in Table 1 below.

The average learning environment data of a typical training session is given below:

- Light intensity: (Not measured due to set up limitation);
- Level of CO_2_: 466.89 PPM;
- Humidity: 45.06%RH;
- Temperature: 25.06 °C

**Figure 6:**
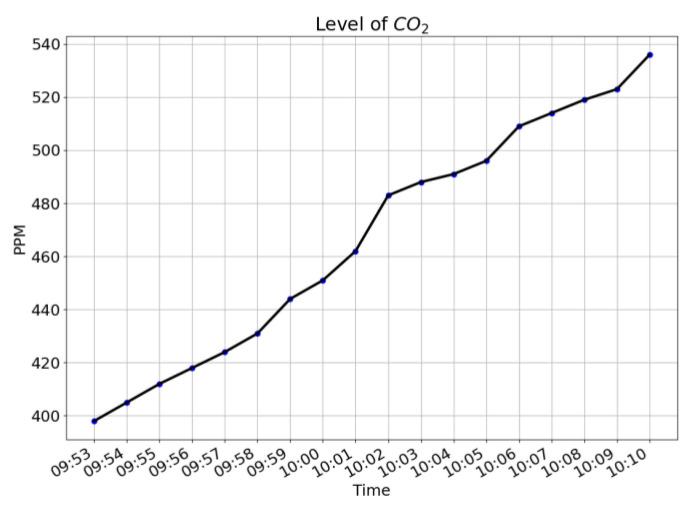
Plot of level of CO_2_ along the timeline of a single training session. An increasing trend is shown.

**Figure 7:**
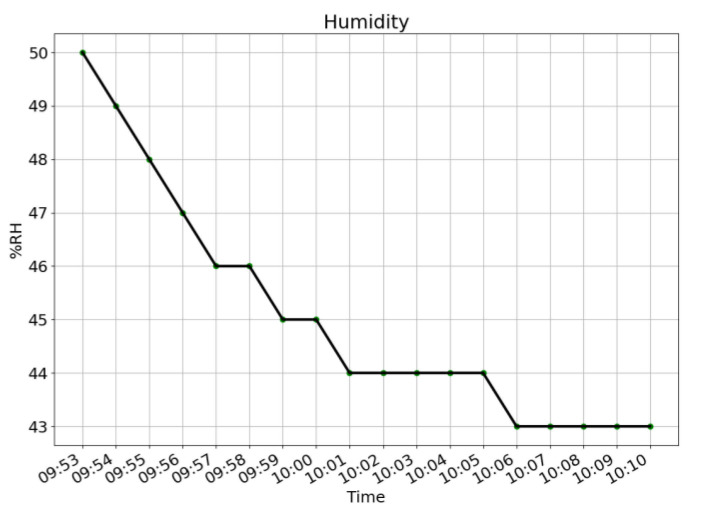
Plot of humidity along the timeline of a single training session. A decreasing trend is shown.

**Figure 8:**
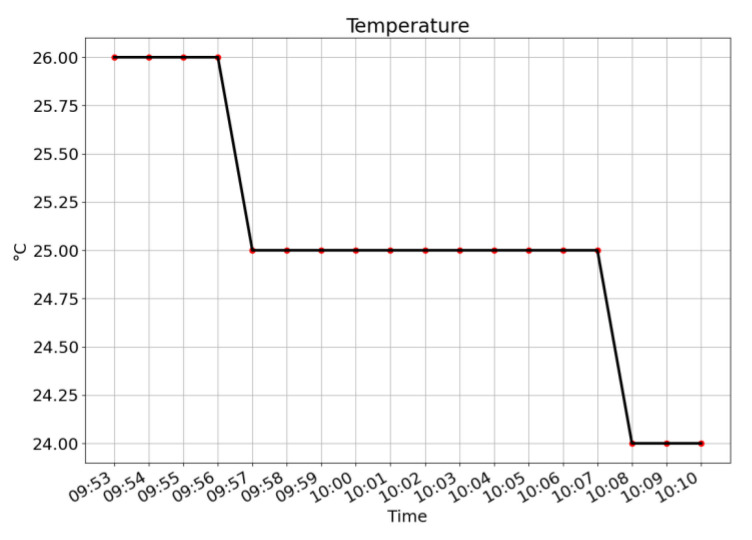
Plot of temperature along the timeline of a single training session. A decreasing trend is shown.

Figure 6, Figure 7 and Figure 8 give the plots of CO_2_ level, humidity, and temperature along the timeline of a single typical training session. For the level of CO_2_, a steadily increasing trend is shown. For both humidity and temperature, steadily decreasing trends are shown.

### 4.2. Students Physiological Data

**Figure 9:**
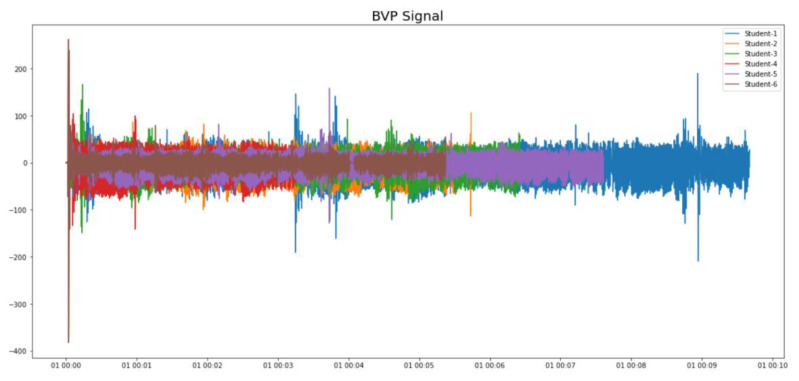
Diverse BVP signals from different students.

**Figure 10:**
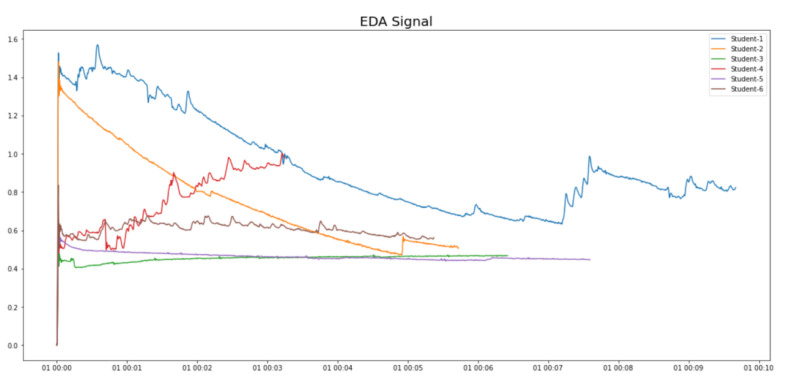
Diverse EDA signals from different students.

We collected physiological data from 15 SEN students (all having ASD and moderate intellectual disabilities). A diverse range of the students’ physiological data was found. For example, the BVP signals and the EDA signals of six of the students are shown in Figure 9 and Figure 10, respectively. It is clear that the physiological data are highly personalized. Therefore, single-subject research [35] (which is a common research practice in SEN research), instead of conventional big data analytics, was suggested for data analysis.

### 4.3. Single Subject Analysis of Individual Student

**Figure 11:**
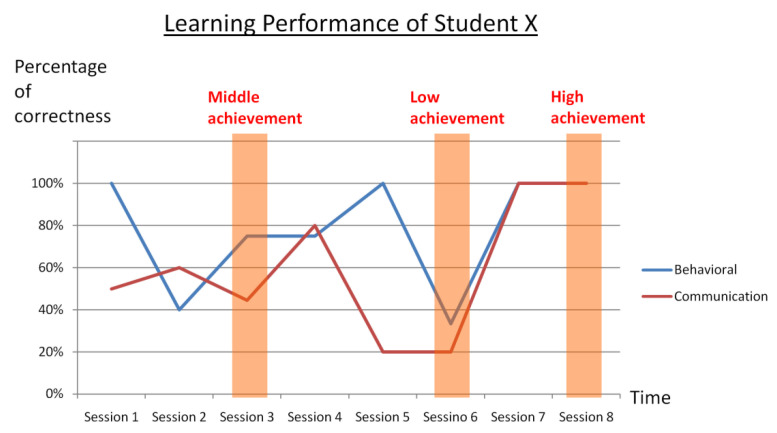
Detailed learning performance of a single student. Low, middle, and high level of achievement occurred in session 6, session 3, and session 8, respectively.

In this section, we look into the physiological data of a single student in detail and attempt to identify any relationship between the student’s learning performance (Figure 11) and the physiological trends.

**Figure 12:**
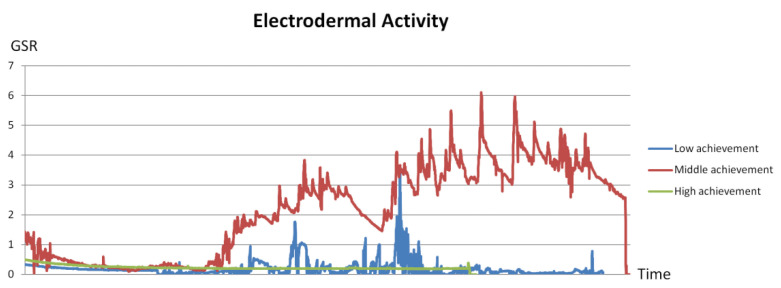
Comparison of electrodermal activity (measured in terms of GSR) of the same subject in (1) low achievement, (2) middle achievement, and (3) high achievement sessions.

In particular, the electrodermal activity (EDA signals, measured in terms of galvanic skin response, GSR) of the same subject collected from session 6 (low achievement), session 3 (middle achievement), and session 8 (high achievement) are compared in Figure 12 below.

As reflected from the evidence, when the student was within the high achievement level, his/her electrodermal activity remained steady and low. Fluctuations are shown when he/she was within middle or low achievement levels. This suggests a possible relationship between the GSR and students’ performance, and requires further investigation.

## 5. System Evaluation with Real Target Users

### 5.1. Participants, Method, and Procedures

A user study was carried out in a 10-week period from May 2021 to July 2021 using the 3i-learning system described above. During the study, ABA therapy sessions were conducted, and the students’ performance was recorded. There were 15 participating students (11 males, 4 females). All students were diagnosed with moderate intellectual disabilities and ASD. The distribution of academic levels of the participating students is given in Table 2 below.

Each of the training sessions lasted for 30 min and were conducted in a 1-to-1 style between a student and a teacher. A personalized Individualized Education Plan (IEP), which is the usual practice in special education, was developed for every participating student, which included training tasks to help them acquire a certain new skill. In each session, the teacher selected 1 to 2 training tasks from the student’s IEP to conduct the practice. Once the student reaches mastery for a learning task, another task at a higher level will be conducted.

### 5.2. Learning Objectives, Scoring, and Assessment Criteria

The learning objectives in ABA therapy are measured in terms of whether a student can perform a task by him or herself without any prompts from the teacher [1]. In our user study, task performances were scored in the form of a percentage of correctness, which was calculated by counting the number of times that the student successfully completed the training task and the total number of times which the task was practiced throughout the session. To reach mastery of a training task, the student needed to obtain a score of over 80% for 3 consecutive sessions, or a score of over 90% for 2 consecutive sessions.

In addition to the students’ ABA performance, the 10 participating teachers were invited to evaluate their perceptions of classroom interventions in terms of (1) acceptability, (2) effectiveness, and (3) time of effect.

### 5.3. Evaluation Result I: Students’ ABA Participation and Performance

A total of 50 training tasks were practiced across the 15 students throughout the entire user study period. The average number of sessions attended by the students was 8.9, with a maximum of 10 sessions and a minimum of 6 sessions. The distribution of the types of training tasks is given in Table 3 below. 3 out of the 50 training tasks were only conducted for a single session; thus, they were not taken into consideration when analyzing the effect of ABA therapy on learning and skill acquisition.

Among the 15 participating students, 13 of them (86.7%) mastered at least 1 training task in their ABA sessions. Among the 47 training tasks being analyzed, 28 of them (59.6%) had the mastery criteria mentioned in Section 5.2 met. On average, each of these 28 mastered training tasks took 3.86 sessions to reach mastery, which equals a little less than 2 h of lesson time.

Looking at the 19 training tasks that did not have the mastery criteria met, 8 of them still showed improvement when comparing the scoring percentages of the first to the last sessions where the task was conducted, which still indicates some positive learning progress. Taking all 47 training tasks into consideration, the score of the tasks had an average improvement of 5 points in each session, showing a generally positive trend in the learning progress for every session that a training task was conducted.

Overall, a majority of the students (13 out of 15) demonstrated progression in learning by mastering at least 1 training task which were skills that the students did not possess previously. The improvement on the training tasks conducted was remarkable, with 76.6% of the conducted training tasks showing a positive learning progress and 59.6% of them reaching mastery. Even with a short session time of 30 min, all training tasks conducted demonstrated an average of 5 points of improvement for each session conducted, suggesting the possibility of an even more remarkable improvement if students can receive ABA therapy for a longer time.

### 5.4. Evaluation Result II: Sensor Readings and Learning Status

Several statistically significant relationships between the learning environment, the learner’s physiological status, and the learning performance have been identified. For example, the level of CO_2_ is correlated to a negative learning performance. In particular, the number of prompts (meaning that the students could not perform a task by him or herself alone, but required the teachers’ prompting) is positively and significantly correlated to the CO_2_ level (*r* = 0.143, *p* < 0.05). The number of incorrectly performed tasks is positively and significantly correlated to the CO_2_ level (*r* = 0.195, *p* < 0.01), too. It was also found that the ambient classroom temperature is significantly and positively correlated to the students’ GSR (*r* = 0.355, *p* < 0.001), also in addition to their skin temperature (*r* = 0.325, *p* < 0.001). Since a few previous studies have attempted to use GSR as a biomarker for students’ performance, e.g., [36], our preliminary findings offer further insights into this direction, especially in SEN settings.

### 5.5. Evaluation Result III: Teachers’ Acceptance

A total of 10 teachers who participated in the user study completed the Behavior Interventions Rating Scale (BIRS) [37], which was an educational research instrument developed to measure teachers’ beliefs regarding educational practice acceptability and their perceived effectiveness of such interventions. The BIRS is a 24-item validated measure of three factors in teachers’ perceptions of classroom interventions: (1) Acceptability, e.g., most teachers would find this intervention suitable for the students’ behaviors; (2) Effectiveness, e.g., the intervention would produce a lasting improvement in the students’ behaviors; and (3) Time of Effect, e.g., the intervention would quickly improve the students’ behaviors. The 6-point Likert-type scale in the questionnaires ranged from 1(strongly disagree) to 6 (strongly agree).

**Figure 13:**
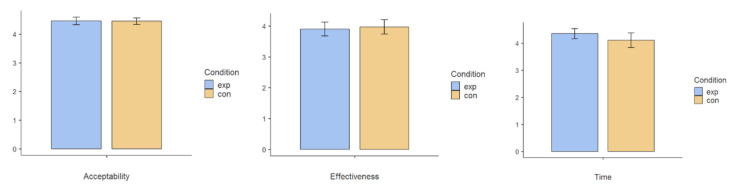
Comparison of participating teachers’ acceptance of the system in terms of (1) acceptability, (2) effectiveness, and (3) time of effect. No significant difference was found between the experimental and control conditions.

After experiencing the 10-week user study intervention period, each of our 10 participating teachers was required to compare their views on conducting IoT-enhanced ABA sessions using the system (experimental condition) and conducting conventional teaching without any technology (control condition). The mean scores were around 4–5, indicating agreement with the acceptability, effectiveness, and time of effectiveness in both intervention approaches (Figure 13). Teachers rated the 2 types of approaches as comparable, with no statistical difference in any factor, which reflects that the new technology was acceptable to the teachers in a way that was comparable to their conventional practices.

Two focus group interviews were conducted with the participating teachers. In general, the teachers agreed that the IoT-enhanced ABA therapy was effective, especially in the later sessions during the user study period, when students had gotten used to the ABA model and showed high attention and an ability to follow directions. The majority of the teachers supported the use of the system in helping their teaching. They agreed that the ABA training sessions using the system were valuable. Some teachers were mindful of damaging the IoT equipment and expressed that they needed guidelines for encountering equipment malfunctions.

## 6. Discussion

### 6.1. On the Performance of Our Current System

Various internal and external factors could influence ABA intervention outcomes in naturalistic settings. The 3i-learning system can collect a multitude of task performance, physiological, and environmental measures that can be analyzed to address initial design goals. Firstly, this study demonstrated the feasibility of having caregivers and teachers conduct ABA therapy sessions at home or at school, and the 3i-learning system is positioned to provide sufficient assistance to users through the utilization of a training tasks database, and AI and IoT measurements. On the other hand, the input of time and resources required from parents and teachers are lowered with the help of technology, as the AI engine is able to provide clinically sound suggestions when it comes to forming an Individualized Education Plan (IEP) for students with SEN. With the aid of IoT sensors for creating SCLEs for students, users can gain a very comprehensive view on factors that are potentially affecting the student’s learning, allowing their progress to be maximized through making crucial adjustments in the learning environment.

The 3i-learning system will still need certain enhancements to reach a higher limit for bringing quality ABA therapy to the majority of students. First of all, further advancements will be needed for our emotion recognition module to include a larger variety of emotion labels, as well as to provide more insight into the intensity of emotion experienced by the students. On top of that, enhancement of the AI engine will also be needed to integrate all the data collected from IoT measurements, to provide more comprehensive suggestions on adjustments and to improve the learning environment and the mood of the students. Furthermore, the cost of the Empatica E4 wristband is considerably high; thus, the development of a wristband specialized for students with SEN with a lower cost will be needed for the system to be applicable to most, if not all, SEN families.

### 6.2. On the Relationship between IoT Sensor Data and Students’ Performance

In this work, we have demonstrated how to apply various IoT sensing technologies to obtain environmental data (temperature, humidity, light intensity, and CO_2_ concentration) and students’ physiological data (blood volume pulse, acceleration, electrodermal activity, and skin temperature) during their ABA therapy. Our exercise enabled us to collect valuable, intensive data about the learner, learning environments, and the learning content, and performed basic statistical analyses on the sensors’ data. As a follow-up work, a structural equation model (SEM) [38]—which is a statistical methodology for the quantification and testing of substantive theories, and explicitly takes measurement errors into account—can be constructed and tested so as to find out any statistical relationship among the learner, learning environment, and learning content variables. Such findings can inform any future use of IoT in the enhancement of ABA therapy, as well as other sensor technology-enhanced SEN practices. The following quantitative relationships will be of interest to future researchers:

- To what extent can the IoT variables/emotion status help increase SEN students’ overall learning outcomes?
- What kinds of emotions are statistically linked to the improvement of SEN students’ learning outcome?
- Which physiological measures predict intervention success as measured by task performance?
- How do environmental variables (light, noise, CO_2_, humidity) affect the physiological measures of children with ASD during the intervention process?

Overall, we have demonstrated the feasibility of creating a data collection system and installing it in a real school classroom. Further follow-ups also include the training of school personnel and teachers who will implement ABA-based therapy in a school setting, so as to inform the creating and tailoring of system features for parents or caregivers.

### 6.3. Limitations

According to a recent review on ABA therapy for autism [39], there were a few existing cases where technologies were applied in ABA practices. These include the use of robots [40,41], avatars [42], computer-based didactic software [43], and augmented reality [44]. There is no formal evaluation standard in ABA technologies so far. Therefore, in the current study, we did not compare our system performance with the few existing technologies mentioned.

## 7. Conclusions

In this paper, we have presented the theoretical background of ASD, ABA, and introduced our 3i-learning system. We have also illustrated how various IoT sensors have been introduced to our system to collect data that are useful for understanding how students went about their ABA therapy. Our evaluation results suggest that the system is effective for students’ skill mastery, and leads to a positive behavioral change. We have also identified a number of statistically significant relationships between the IoT sensor measurements and learning-related indicators. Also, our teacher survey demonstrated an appreciable teacher acceptance of our system. To conclude, through providing sufficient training and utilizing the 3i-learning system, the cost of therapy can be brought down significantly while allowing SEN children to be engaged in intensive ABA therapy. On the other hand, parents and caregivers will be better equipped to interact and foster their children, while giving them a consistent environment to learn and develop. Through working with potential users and collaborating with schools and Non-Governmental Organizations (NGOs), the 3i-learning system will bring quality behavioral therapy to families with children with SEN, especially underprivileged ones, and ultimately promote a harmonious society which welcomes individuals with SEN.

## Figures and Tables

**Table 1:** Summary of learning environment data.

Parameter Name	Recording Device	Units
Light Intensity	REEDSD-9300 Data Logging Environmental Meter	LUX
Level of CO_2_	GZAIR Model 2 Indoor CO_2_ Meter	PPM
Humidity	REED SD-9300 Data Logging Environmental Meter	%RH
Temperature	REED SD-9300 Data Logging Environmental Meter	Degree Celsius

**Table 2:** Academic Level of Participating Students.

Academic Level	Number of Participants
Junior Primary	7
Senior Primary	2
Junior Secondary	6
Senior Secondary	0

Note: Chronological age of the students ranged from 7 to 16 years old.

**Table 3:** Domain and Distribution of Training Task Practiced in the User Study.

ABA Training Task Domain	Number of Tasks Practiced
Communication	19 (38%)
Behavior Development	16 (32%)
Academic & Learning	13 (26%)
Independence & Self-help	2 (4%)
Total	50 (100%)

## Data Availability

Anonymized data will be made available on request to the correspondent author’s email with justification.
